# Supplementary material for: Problematic Social Media Use and Depressive Outcomes among College Students in China: Observational and Experimental Findings
Source: Int J Environ Res Public Health. 2022 Apr 19;19(9):4937. doi: 10.3390/ijerph19094937 (PMC9099455; doi:10.3390/ijerph19094937)

Supplemental Table S1. The detail of sampling colleges and students of the cross-sectional study (Study 1)

Number	College type	College names	Freshman	Sophomore	Junior	Senior	Graduates	Total
1	Local university	Xi’an Jiaotong University	200	200	200	100	100	800
2	Local university	Northwest A&F University	200	200	200	100	100	800
3	Local university	Shaanxi Normal University	200	200	200	100	100	800
4	Local university	Chang’an University	200	200	200	100	100	800
5	College directly under the Ministry of Education	Northwest University	200	200	200	100	100	800
6	College directly under the Ministry of Education	Shaanxi University of Science & Technology	200	200	200	100	100	800
7	College directly under the Ministry of Education	Yan'an University	200	200	200	100	100	800
8	College directly under the Ministry of Education	Xi`an Polytechnic University	200	200	200	100	100	800

9	College directly under the Ministry of Education	Northwest University of Politics & Law	200	200	200	100	100	800
10	College directly under the Ministry of Education	Shaanxi University of Technology	200	200	200	100	100	800
11	College directly under the Ministry of Education	Xi`an Medical University	200	200	200	100	0	700
12	College directly under the Ministry of Education	Baoji University of Arts and Sciences	200	200	200	100	0	700
13	College directly under the Ministry of Education	Weinan Normal University	200	200	200	100	0	700
14	College directly under the Ministry of Education	Ankang University	200	200	200	100	0	700
15	College directly under the Ministry of Education	Shangluo College	200	200	200	100	0	700
16	College directly under the Ministry of Education	Yulin University	200	200	200	100	0	700
17	Private university	Xi`an Peihua University	200	200	200	100	0	700

18	Private university	Xi`an Eurasia University	200	200	200	100	0	700
19	Private university	Xijing University	200	200	200	100	0	700
20	Private university	Xi'an Siyuan university	200	200	200	100	0	700
21	Vocational university	Yangling Vocational & Technical College	300	200	100	0	0	600
22	Vocational university	Shaanxi Vocational and Technical College	300	200	100	0	0	600
23	Vocational university	Shanxi vocational college of finance and economics	300	200	100	0	0	600
24	Vocational university	Xi'an Aeronautical Polytechnic Institute	300	200	100	0	0	600

25	Vocational university	Baoji Vocational Technology College	300	200	100	0	0	600
26	Vocational university	Xianyang Vocational Technical College	300	200	100	0	0	600
27	Vocational university	Tongchuan Vocational Technical College	300	200	100	0	0	600
28	Vocational university	Weinan Vocational Technical College	300	200	100	0	0	600
29	Vocational university	Yanan Vocational Technical College	300	200	100	0	0	600
30	Vocational university	Hanzhong Vocational	300	200	100	0	0	600

		Technical College						
Total number	30		7000	6000	5000	2000	1000	21000

Supplemental File S1. Calculations for determining sample size of the intervention study (Study 2) among college students in Shaanxi, China

In this study, the following formula 1 was used to calculate the sample size required for the intervention and control group for the intervention trial. We carried out a pilot study of an intervention for depression in college students. According to the results of the pilot study and findings of previous studies (Li, et al., Chinese Journal of Health Psychology, 2014, 22), after 5 weeks of depression intervention among college students college students, the between-group difference of change on depressive symptoms was 15.47, and the standard error of the mean before and after the intervention in the intervention group and control group were 12.26 and 5.26, respectively.α=0.05, β=0.1, Z_{α}=1.96, Z_{β}=1.96, PASS was used to calculate the sample size. After the calculation, at least 11 college students were needed in each group, so the total sample size was at least 22. To improve the power of the intervention trial, 30 samples were recruited for the intervention group and the control group, with a total sample size of 60 in the RCT.

$$\mathbf{N} = \frac{(\mathbf{Z}_{1-\alpha/2} + \mathbf{Z}_{1-\beta})^2 \times (\boldsymbol{\sigma}_1^2 + \boldsymbol{\sigma}_2^2)^2}{\boldsymbol{\delta}^2}$$

Supplemental Table S2. Details of the intervention activities for depressive symptoms in the intervention group of college students in Shaanxi, China

Intervention stage	Intervention theme	Intervention time	Intervention means	Intervention content
Stage 1	The effect of social network use on depressive symptoms	2.5 hour	Offline instruction	1) Introduce current use of social network among college students;<br>2) Introduce basic situation of depression in college students;<br>3) Emphasize the importance of good interpersonal relationships;<br>4) Introduce the effects of social network addiction on depressive symptoms.
Stage 2	Self-cognition remodeling based on the effect of social network use on depressive symptoms	2.5 hour	Online instruction	1) Ask students to draw a self-portrait on A4 paper and name it;<br>2) Introduce Satir Transformational Systemic Therapy；<br>3) Introduce the iceberg theory.
Stage 3	Time management group counseling based on self-cognition	3.5 hour	Offline group counseling	1) Play ice breaking game the wind blows;<br>2) Map and analyze of personal lifelines;<br>3) Share ideal time allocation pie chart;<br>4) Play a thousand knots in heart activity.

Stage 4	Network interpersonal communication skills training based on self-cognition	2.5 hour	Online instruction	1) Introduce individual communication purpose and interpersonal communication forms;<br>2) Introduce network interpersonal communication platform features;<br>3) Introduce common network interpersonal communication problems;<br>4) Introduce network interpersonal communication skills.
Stage 5	Group counseling for the improvement of network and real interpersonal relationships based on self-cognition	3.5 hour	Offline group counseling	1) Play incubating egg game;<br>2) Make and introduce personal business cards;<br>3) Play Russian turntable, role-playing and transformation;<br>4) Draw and analyze of personal interpersonal circle;<br>5) Play happy massage circle, truly understand the importance of human relations.

Supplemental Figure S1. The course of intervention among college students in Shaanxi, China

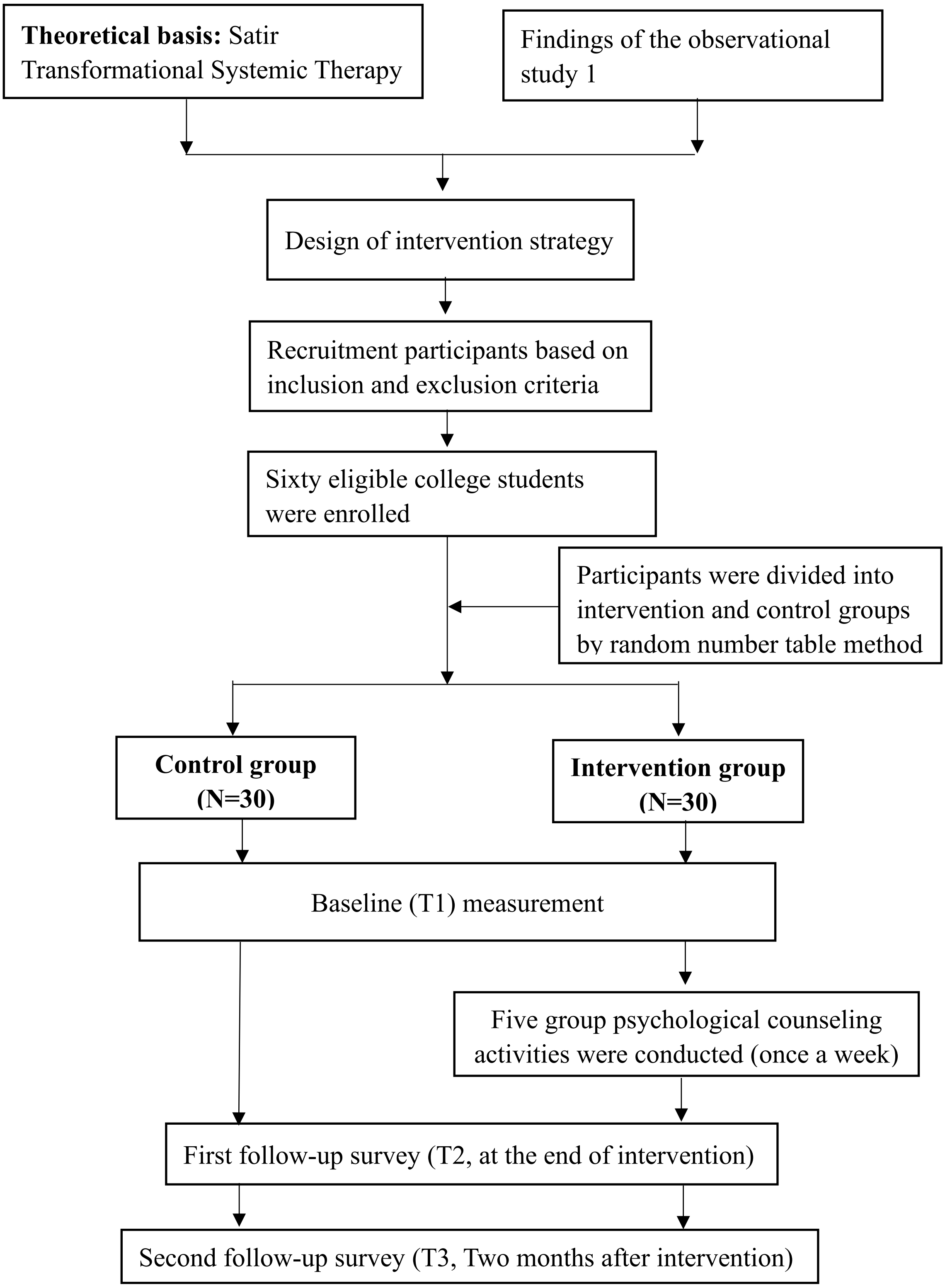

Supplement: Supplementary file 1 [file ijerph-19-04937-s001.zip › ijerph-1627750-supplementary.pdf]
